# External validation and application of machine learning models for obesity in Chinese children and adolescents per Chinese and WHO growth standards

**DOI:** 10.7189/jogh.16.04299

**Published:** 2026-10-19

**Authors:** Fangjieyi Zheng, Xiaoqian Wang, Mei Xue, Qiong Wang, Wenqian Zhang, Zhixin Zhang, Wenquan Niu

**Affiliations:** 1 Center for Evidence-based Medicine, Capital Center for Children’s Health, Capital Medical University, Capital Institute of Pediatrics, Beijing, China; 2 Chinese Academy of Medical Sciences & Peking Union Medical College, Beijing, China; 3 Graduate School, Beijing University of Chinese Medicine, Beijing, China; 4 Department of Pediatrics, China-Japan Friendship Hospital, Beijing, China; 5 Institute of Clinical Medical Sciences, China-Japan Friendship Hospital, Beijing, China

**Keywords:** children and adolescents, Chinese and WHO growth standards, external validation, machine learning, obesity

## Abstract

**Background:**

Childhood and adolescent obesity is a growing global public health challenge, underscoring the need for reliable tools to identify individuals at high risk. We aimed to develop, validate, and deploy machine learning (ML) models for predicting obesity in children and adolescents aged 3–18 years using multi-domain risk factors per Chinese and World Health Organization (WHO) growth standards, with an online tool for clinical and public health translation.

**Methods:**

We conducted a population-based cross-sectional study. We used data from 22,555 participants in Beijing (2022, 2024) for training/internal validation, and data from 5,343 participants in Tangshan (2020) for external validation. We defined obesity using both Chinese and WHO growth standards. We analysed 38 features across 5 domains; we selected key predictors using least absolute shrinkage and selection operator regression. We evaluated 4 ML models using 28 performance metrics, with SHapley Additive exPlanations enhancing interpretability.

**Results:**

We retained 5 features per the Chinese growth standards and 12 per the WHO growth standards. Age, maternal body mass index (BMI), paternal BMI, screen time, and birthweight were consistent predictors. The neural network achieved optimal performance per Chinese growth standards (area under the receiver operating characteristic curve (AUROC) = 0.735), and eXtreme gradient boosting performed best per WHO growth standards (AUROC = 0.736), with the corresponding external validation AUROCs of 0.699 and 0.707. We subsequently deployed user-friendly online prediction tools for translation.

**Conclusions:**

We identified two interpretable ML models for obesity in children and adolescents per both Chinese and WHO growth standards, each demonstrating modest but reliable predictive performance.

Obesity in children and adolescents has surged to epidemic proportions, posing a major global public health challenge and imposing substantial burdens on healthcare systems [1]. Recent meta-analyses have indicated that the global obesity prevalence of children and adolescents was 8.5%, representing a 1.5-fold rise between 2012 and 2023 compared to the 2000–2011 period [2], with marked cross-country heterogeneity (0.4% in Vanuatu to 28.4% in Puerto Rico). Globally, the age-standardised prevalence of obesity among children and adolescents aged 5–19 years was 7.8% in boys and 5.6% in girls in 2016, based on WHO criteria [3]. Obesity in children and adolescents often persists into adulthood [4,5], exerting significant adverse effects on cardio-metabolic and respiratory health [6–8]. As a largely preventable condition, early intervention targeting modifiable risk factors represents the most effective strategy to address this global public health challenge. Thus, a comprehensive understanding of risk profiles underlying obesity in children and adolescents is critical for informing targeted preventive and interventional strategies.

The World Health Organization (WHO) obesity definition standards provide a universal reference for cross-population comparisons [9]; however, most countries, including China, have established their own national growth references. The lack of unified diagnostic cut-offs compromises consistent obesity assessment in public health and clinical settings [10–12]. In December 2024, China’s National Health Commission released updated weight management guidelines [13] that aim to better capture the physical growth patterns of Chinese children and adolescents. By nature, the WHO and Chinese growth standards differ in statistical methodologies and the underlying reference populations. Consequently, obesity prevalence estimates and individual classification results vary considerably between the two systems, especially for children and adolescents approaching diagnostic thresholds. These inconsistencies yield uncertainties in estimating the actual burden of paediatric obesity and hinder the identification of key risk factors when evaluated under different diagnostic frameworks.

A wide range of modifiable factors have been linked to obesity in children and adolescents. Population-based cross-sectional studies from Pakistan and Ethiopia have identified sleep duration, maternal age, household socioeconomic status, fuel type, and mobile phone ownership as independent predictors of overweight and obesity [14,15]. Additional risk factors include ultra‑processed food intake, maternal junk food consumption, rapid eating speed, and late bedtime [16–18]. Despite these findings, the specific determinants of paediatric obesity and their relative contributions remain incompletely understood, partly due to its multifactorial aetiology. Most prior studies have focused on individual or single‑domain risk profiles [19,20] often overlooking their potential additive and synergistic interactions; moreover, insufficient sample sizes in some studies have limited statistical power to detect modest or small effects. Traditional statistical approaches, including linear regression and logistic regression, have been widely used to identify obesity‑related factors [11]. However, these approaches rely on strict assumptions of independence and linearity that are frequently violated in real‑world datasets, potentially yielding unreliable or misleading conclusions [21,22]. To address these limitations, large homogeneous cohorts and advanced statistical algorithms are needed to integrate multi‑domain risk profiles and characterise their complex, nonlinear, and high‑dimensional interactions.

Since 2017, our team has conducted six waves of regional cross-sectional surveys among Beijing’s children and adolescents aged 3–18 years, gathering multi-domain data from both participants and their parents/guardians [23–30]. Leveraging data from the two most recent waves, we adopted machine learning (ML) algorithms to explore the association between multi-domain factors and obesity risk per both Chinese and WHO growth standards. Specifically, we optimised model performance *via* hyperparameter tuning and cross-validation, identified the minimal sufficient feature set *via* regularisation, validated the best-performing model using SHapley Additive exPlanations (SHAP), and finally deployed the validated model as an online prediction tool to facilitate clinical and public health translation.

## METHODS

### Study design and participants

We derived training and internal validation data from two waves of our ongoing series of regional cross-sectional surveys conducted in Pinggu district, Beijing, China. The first wave was implemented between January and February 2022, and the second between February and June 2024. Both waves adopted a systematic stratified cluster sampling strategy to recruit children and adolescents aged 3–18 years enrolled from kindergartens, primary schools, and middle schools. This sampling strategy ensures representative coverage across different strata and clusters of educational institutions. The 2022 wave included participants from 8 primary schools and 18 middle schools, while the 2024 wave expanded recruitment to include 7 kindergartens, 7 primary schools, and 3 middle schools. External validation data were derived from our prior survey in Tangshan city, Hebei province, China, conducted in 2020, including 5,343 children and adolescents aged 3−18 years.

We obtained written informed consent from the parents or guardians of all participating children and adolescents before participation. To ensure confidentiality, we anonymised survey data and assigned unique identifiers for tracking purposes.

### Data collection

We collected data through self-designed electronic questionnaires distributed *via* the Wenjuanxing platform [31] and on-site measurements (weight and height). Before formal circulation, we assessed the reliability and validity of the questionnaires through a pilot study with 200 participants, and the internal consistency was acceptable (Cronbach’s alpha coefficients > 0.85).

### Quality control

Prior to data collection, designated health practitioners and teachers from participating kindergartens and schools completed training on survey procedures and questionnaire content. During data collection, these trained personnel assisted parents or guardians as needed. All identified missing or implausible values were verified *via* direct follow-up with parents or guardians to ensure data accuracy.

### Obesity definition

Trained health practitioners and school staff measured body height and weight to the nearest 0.1 cm and 0.1 kg, respectively, using pre-mailed measuring devices to ensure consistency and reduce measurement error. We calculated body mass index (BMI) by dividing weight (kg) by height (m^2^).

We defined obesity status per both WHO growth standards and the Chinese growth standards established by the Chinese National Health Commission in December 2024. The WHO growth standards rely on age- and sex-standardised BMI z-scores, which express the deviation of a participant’s BMI from the reference population mean in standard deviation (SD) units. The 2006 WHO child growth standards were applied for children <5 years; the 2007 WHO growth references are used for those aged 5–19 years (61–228 months) [9]. Under the WHO framework, obesity is defined as a BMI z-score > 2 SD.

The Chinese growth standards also use age- and sex-specific BMI z-scores for children >7 years, with obesity similarly defined as a z-score > 2 SD. However, the two frameworks differ in age-stratification intervals: the WHO uses six-month intervals, and the Chinese standards use three-month intervals. For children and adolescents aged 7–18 years, the Chinese growth standards used fixed age- and sex-specific BMI cutoff points to define obesity [13].

### Feature definition

We categorized questionnaire items into five domains: demographic characteristics (date of birth, age, and sex), foetal and early-life factors (gestational age, mode of delivery, pregnancy and delivery order, twin birth, birth length and birthweight, infancy feeding practices including breastfeeding duration and time to solid food introduction), daily lifestyle behaviours (sedentary time, screen time, outdoor physical activity duration, bedtime, eating speed, weekly intake frequencies of sweetened foods, late-night snacks, and fast food), health conditions (history of food or drug allergies, number of dental caries, and presence of chronic diseases), and family information (household income, parental height and weight, parental education attainment, and parental age at childbirth). Item selection was guided by existing literature and expert consensus in paediatric obesity research.

Consistent with ML terminology, we referred to all variables derived from questionnaires and anthropometry as features (Appendix S1 in the **Online Supplementary Document**).

### Statistical analyses

To rigorously prevent data leakage, we randomly partitioned the full dataset (two waves) into a training subset (n = 11,362) and an internal validation subset (n = 7,574) at a 6:4 ratio. We performed subsequent data-adaptive preprocessing, feature selection, and model tuning exclusively on the training subset, with derived parameters applied blindly to the validation subset. We performed feature preprocessing in seven sequential steps. First, we filtered missingness, excluding features with >30% missingness. Second, we handled outliers using the Tukey method [32] (*i.e.* values < Q1 – 1.5 × interquartile range (IQR) or > Q3 + 1.5 × IQR were flagged as missing). Third, missing data imputation: we generated five imputed datasets using multiple imputation; each ML model was independently fitted on all five datasets; final predictive performance (area under the receiver operating characteristic curve (AUROC)) for each model was pooled by averaging across the five replicates to account for imputation uncertainty. Fourth, we assessed correlations; for highly correlated feature pairs (absolute Spearman’s ρ ≥ 0.8), we removed the feature with lower clinical relevance or weaker association with obesity. Fifth, we assessed multicollinearity and removed features with variance inflation factor > 5. Sixth, we standardised continuous features using z-scores to ensure uniform scaling across models. Lastly, we applied least absolute shrinkage and selection operator (LASSO) regression with L1 regularisation for feature selection to reduce dimensionality and minimise overfitting while retaining interpretable predictors.

We trained and internally validated four ML models: K-nearest neighbours (KNN), Light Gradient Boosting Machine (LightGBM), a neural network, and eXtreme Gradient Boosting (XGBoost). We selected the models based on their established performance in biomedical prediction tasks and ability to model complex non-linear associations. Of the four ML models, KNN is a non-parametric, instance-based classifier; LightGBM uses histogram-based boosting to improve efficiency; neural networks capture high-dimensional non-linear relationships; and XGBoost integrates regularisation to control overfitting while maintaining computational speed. We performed hyperparameter tuning *via* grid search with 30 parameter combinations and five-fold cross-validation [33,34].

We evaluated model performance for binary classification (obesity *vs.* normal weight) using core clinically meaningful metrics, including AUROC, accuracy, balanced accuracy, precision, F-beta score, sensitivity, specificity, binary Brier score, calibration intercept, and calibration slope. We also calculated the Matthews correlation coefficient, predictive values, and diagnostic odds ratio (Table S1 in the **Online Supplementary Document**).

We enhanced model interpretability using SHAP, a game theory-based framework that quantifies the marginal contribution of each feature to individual predictions. We applied TreeSHAP for tree-based models (LightGBM, XGBoost) and KernelSHAP for KNN and neural network models. Waterfall plots illustrated how each feature shifts the baseline population risk toward a final individual obesity probability. Force plots decomposed predictions into risk-increasing and risk-decreasing components, supporting intuitive interpretation of personalised risk profiles. For clinical translation, we deployed an interactive web-based prediction tool using the R Shiny package. The tool incorporates LASSO-selected features and supports risk stratification per both WHO and Chinese growth standards. Users enter required data in a secure web interface; the server computes personalised obesity probabilities and meanwhile generates participant-specific SHAP waterfall plots. This tool enables clinicians and caregivers to evaluate modifiable risk contributions and supports targeted preventive strategies.

We performed between-group comparisons (obesity *vs.* normal weight) using independent t-tests, Mann-Whitney U tests, or chi-square tests, as appropriate. We presented normally distributed continuous features as mean (SD), skewed features as median (IQR), and categorical features as counts and percentages (%). All statistical tests were two-tailed, with *P* < 0.001 considered statistically significant.

To evaluate model robustness across age and diagnostic criteria (z-scores for younger children *vs.* fixed cutoffs for older children), we reassessed AUROC in three predefined age subgroups (3–6, 7–12, 13–18 years) on the internal validation set.

For external validation, we retrospectively applied the final locked models to an independent cohort of 5,343 children and adolescents aged 3–18 years from Tangshan, Hebei Province, collected in 2020. This cohort was not used for feature selection, preprocessing parameter estimation, hyperparameter tuning, model selection, or threshold determination. This analysis assessed the geographical transportability of the models in a historical external cohort rather than their prospective temporal validity.

We used *R*, version 4.4.3 (R Core Team, Vienna, Austria) for all analyses. We used ‘mlr3’ and ‘mlr3proba’ packages for model construction and ‘kernelshap’ and ‘shapviz’ for SHAP values and visualisations.

## RESULTS

The overall analytical pipeline included three sequential phases: participant eligibility and selection, feature selection and model construction, and model appraisal and deployment (**Figure 1**).

**Figure 1:**
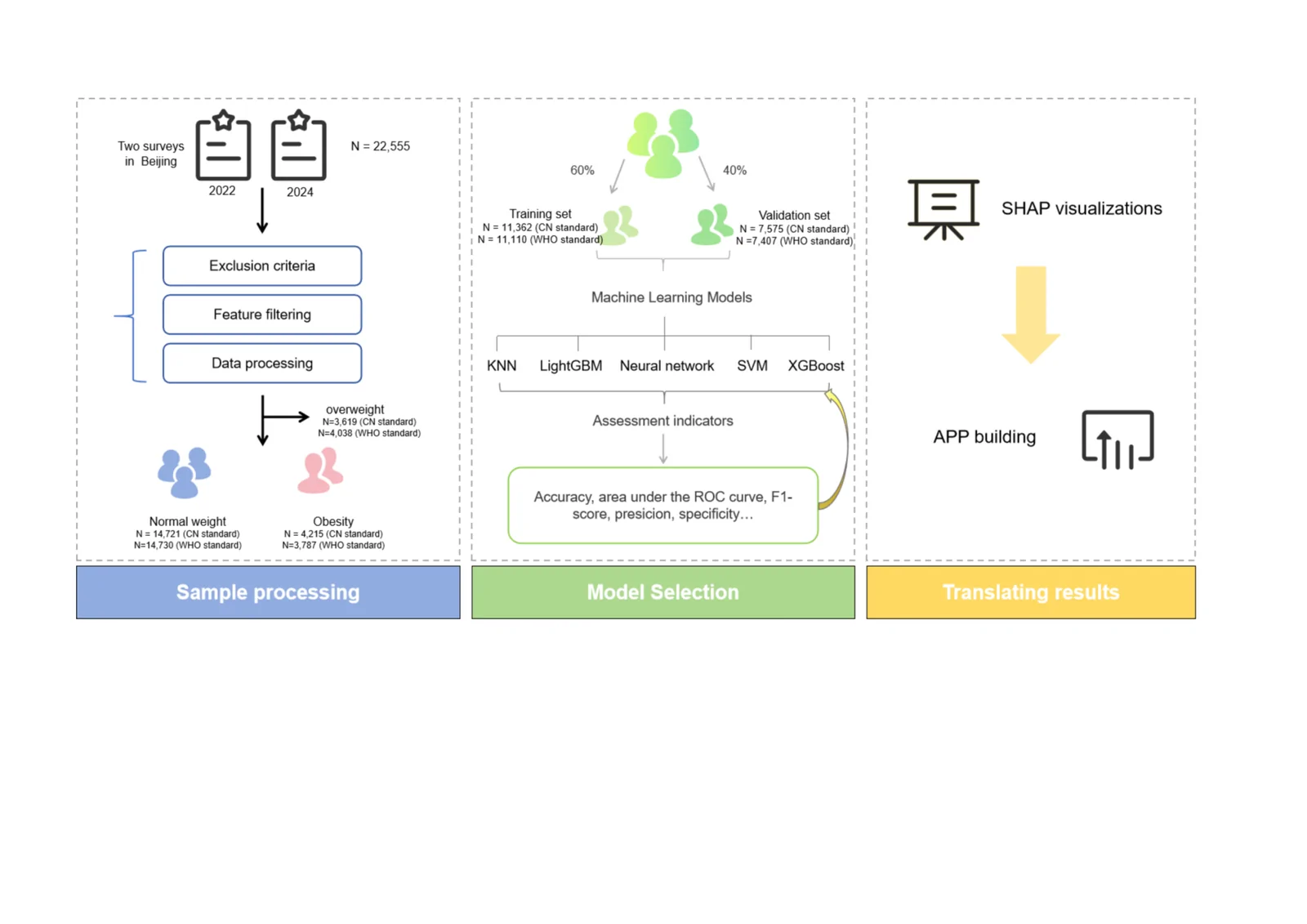
Analytic pipeline of the study. APP – application, KNN – K-Nearest Neighbours Algorithm, LightGBM – light gradient boosting machine, ROC – receiver operating characteristic curve, SHAP – SHapley Additive exPlanations, XGBoost – eXtreme gradient boosting, WHO – World Health Organization.

### Baseline characteristics

We enrolled participants from two waves of our ongoing surveys. After rigorous data cleaning and integration, 22,555 children and adolescents aged 3–18 years were initially eligible. Per Chinese standards, 3,619 participants were classified as overweight, while according to WHO standards, 4,038 were overweight. To improve the signal-to-noise ratio and identify robust predictive features that distinguish clinical obesity from normal-weight development, we excluded participants in the overweight category. After exclusion, the final analytic sample included 18,936 participants (normal weight n = 14,721; obesity n = 4,215) per Chinese growth standards and 18,517 (normal weight n = 14,730; obesity n = 3,787) per WHO growth standards (**Table 1**).

**Table 1:** Baseline characteristics of children and adolescents

	Chinese standard		WHO standard	
	**Obesity (n = 4,215)**	**Normal weight (n = 14,721)**	**Obesity (n = 3,787)**	**Normal weight (n = 14,730)**
**Demographic factors**				
Age in years*	9.33 (6.33–12.17)	7.25 (4.75–11.42)	9.37 (6.41–12.17)	6.83 (4.75–11.17)
Boys†	2,542 (60.31)	7,056 (47.93)	2,596 (68.55)	6,838 (46.42)
Weight, kg*	52.00 (35.00–70.00)	23.00 (17.50–39.00)	50.00 (34.00–76.00)	25.00 (18.00–42.00)
Height, cm*	145 (125–160)	125 (110–152)	156 (115–163)	136 (120–162)
**Foetal and early-life factors**				
Gestational age in weeks*	39 (38–40)	39 (38–40)	39 (38–40)	39 (38–40)
Gravidity*	1 (1–2)	1 (1–2)	1 (1–2)	1 (1–2)
Parity*	1 (1–2)	1 (1–2)	1 (1–2)	1 (1–2)
Delivery mode†				
*Vaginal delivery*	1,793 (42.54)	7,541 (51.23)	1,573 (41.54)	7,575 (51.43)
*Caesarean section*	2,374 (56.32)	7,024 (47.71)	2,170 (57.30)	7,004 (47.55)
*Forceps delivery*	48 (1.14)	156 (1.06)	44 (1.16)	151 (1.03)
Birth length, cm*	50 (50–52)	50 (50–52)	50 (50–52)	50 (50–52)
Birthweight, g*	3,450 (3,100–3,720)	3,300 (3,000–3,600)	3,495 (3,100–3,750)	3,300 (3,000–3,600)
Infancy feeding†				
*Pure breastfeeding*	2,346 (55.66)	8,373 (56.88)	2,095 (55.32)	8,388 (56.95)
*Partial breastfeeding*	1,411 (33.48)	5,007 (34.01)	1,269 (33.51)	5,002 (34.09)
*Non-breastfeeding*	458 (10.87)	1,341 (9.11)	423 (11.17)	1,320 (8.96)
Breastfeeding duration*	11 (0–15)	12 (5–16)	11 (0–15)	12 (6–16)
Time to add solid food*	6.00 (6.00–7.00)	6.00 (6.00–6.50)	6.00 (6.00–7.00)	6.00 (6.00–6.50)
**Lifestyle-related behaviours**				
Screen time in hours per day*	1.29 (0.93–2.29)	1.21 (0.64–2.00)	1.286 (1.00–2.29)	1.143 (0.64–2.00)
Exercise time in hours per week*	1.29 (1.00–2.00)	1.29 (1.00–2.29)	1.286 (1.00–2.00)	1.29 (1.00–2.29)
Sleep time in hours per day*	9.00 (8.29–9.57)	9.28 (8.29–10.00)	9.00 (8.29–9.57)	9.29 (8.29–10.00)
Night meals per week†				
*None or once in a while (1–2)*	741 (17.58)	4,295 (29.18)	583 (15.39)	4,410 (29.94)
*1–3*	1,193 (28.30)	4,418 (30.01)	1,062 (28.04)	4,411 (29.95)
*4–6*	1,619 (38.41)	4,238 (28.79)	1,464 (38.66)	4,140 (28.11)
*Every day*	662 (15.71)	1,770 (12.02)	678 (17.90)	1,769 (12.01)
Bedtime*	9.50 (9.00–10.00)	10.00 (9.00–10.00)	10.00 (9.00–10.00)	9.50 (9.00–10.00)
Eating speed in minutes per meal†				
*<15*	1,583 (37.78)	4,836 (32.99)	1,439 (38.20)	4,848 (33.06)
*15–30*	2,503 (59.74)	9,178 (62.62)	2,231 (59.22)	9,166 (62.50)
*≥30*	104 (2.48)	643 (4.39)	97 (2.57)	652 (4.45)
Fried food intake per week†				
*None or once in a while (1–2)*	554 (13.14)	4,326 (29.39)	473 (12.49)	3,958 (26.87)
*1–3*	1,458 (34.59)	4,047 (27.49)	486 (12.83)	4,458 (30.26)
*4–6*	1,687 (40.02)	3,894 (26.45)	1,527 (40.32)	3,781 (25.67)
*Every day*	1,458 (34.59)	2,454 (16.67)	1,301 (34.35)	2,533 (17.20)
Sweet food intake per week†				
*None or once in a while (1–2)*	867 (20.57)	2,195 (14.91)	783 (20.68)	4,916 (33.37)
*1–3*	990 (23.49)	6,319 (42.93)	891 (23.53)	6,277 (42.61)
*4–6*	2,094 (49.68)	4,837 (32.86)	1,877 (49.56)	2,154 (14.62)
*Every day*	264 (6.26)	1,370 (9.31)	236 (6.23)	1,383 (9.39)
**Health conditions**				
Upper respiratory infections in the past year*	1 (0–2)	1 (0–2)	1 (0–2)	1 (0–2)
Lower respiratory infections in the past year*	0 (0–0)	0 (0–0)	0 (0–0)	0 (0–0)
Number of cavities†	1,686 (40.00)	6,889 (46.80)	1,523 (40.22)	6,917 (46.96)
Asthma†	273 (6.48)	531 (3.61)	256 (6.76)	520 (3.53)
Rhinitis†	1,194 (28.33)	3,287 (22.33)	1,095 (28.91)	3,252 (22.08)
Eczema†	976 (23.16)	3,575 (24.29)	871 (23.00)	3,604 (24.47)
**Family information**				
Maternal reproductive age in years*	27.08 (24.91–29.84)	27.84 (25.42–30.67)	27 (24.83–29.83)	27.92 (25.50–30.75)
Paternal reproductive age in years*	28.17 (26.00–31.00)	28.83 (26.50–32.01)	28.17 (26.00–31.00)	28.84 (26.50–32.10)
Maternal height, cm*	161 (158–165)	161 (159–165)	161 (158–165)	162 (159–165)
Paternal height, cm*	174 (170–178)	175 (170–178)	174 (170–178)	175 (170–178)
Maternal weight, kg*	64.00 (57.00–73.00)	60.00 (53.00–65.00)	64.00 (57.00–73.50)	60.00 (53.00–65.00)
Paternal weight, kg*	80 (74–90)	76 (70–85)	80 (75–90)	76 (70–85)
Maternal BMI, kg/m^2^*	24.34 (21.88–27.77)	22.46 (20.45–24.92)	24.31 (21.88–27.89)	22.46 (20.44–24.89)
Paternal BMI, kg/m^2^*	26.89 (24.49–30.07)	25.24 (23.04–27.76)	27.02 (24.49–30.07)	25.25 (23.03–27.76)
Maternal education attainment†				
*High school degree or below*	1,787 (42.40)	5,504 (37.39)	1,601 (42.28)	5,478 (37.19)
*Bachelor’s degree*	2,334 (55.37)	8,284 (56.27)	2,102 (55.51)	8,307 (56.40)
*Master’s degree or above*	94 (2.23)	933 (6.34)	84 (2.22)	945 (6.42)
Paternal education attainment†				
*High school degree or below*	2,121 (50.32)	6,368 (43.26)	1,889 (49.88)	6,335 (43.01)
*Bachelor’s degree*	1,975 (46.86)	7,322 (49.74)	1,794 (47.37)	7,349 (49.89)
*Master’s degree or above*	119 (2.82)	1,031 (7.00)	104 (2.75)	1,406 (7.10)
Family income in CNY per year†				
*<100,000*	1,773 (44.65)	5,693 (40.44)	1,579 (44.38)	5,680 (40.33)
*100,000–300,000*	1,795 (45.20)	6,073 (43.14)	1,620 (45.53)	6,074 (43.13)
*≥300,000*	403 (10.15)	2,312 (16.42)	359 (10.09)	2,330 (16.54)
Family history of diabetes†	2,357 (55.92)	9,640 (65.48)	2,130 (56.25)	9,785 (66.43)

BMI – body mass index, WHO – World Health Organization

*Values are presented as median (interquartile range).

†Values are presented as n (%).

### Feature selection

We included 37 features with missingness rates < 30%. We handled missing data using multiple imputation and generated five imputed datasets. We standardised all continuous features using z-score transformation to eliminate scale heterogeneity. We randomly partitioned each dataset into training and validation subsets at a 6:4 ratio. We used a fixed random seed of 1,234 to ensure experimental repeatability. During sample partitioning, we fully considered institutional clustering, including kindergartens, primary schools, and middle schools, to maintain balanced obesity prevalence in the training and validation subgroups.

Using LASSO regression, we identified five features for obesity defined per Chinese growth standards, including age, maternal BMI, paternal BMI, screen time, and birthweight. Per WHO growth standards, we retained 12 features: age, maternal BMI, paternal BMI, screen time, birthweight, paternal age at conception, gestational age, outdoor physical exercise duration, complementary feeding time, breastfeeding duration, birth length, and paternal weight (Figure S1 in the **Online Supplementary Document**).

### Model selection and performance

We trained four ML models (KNN, LightGBM, neural network, XGBoost) on the training subset and evaluated them on the validation subset within each imputed dataset. We selected the dataset with the highest average cross-imputation AUROC for downstream analyses. We assessed model performance using multiple metrics for obesity under both Chinese and WHO growth standards (**Figure 2**; **Table 2**). For Chinese growth standards, the neural network achieved optimal predictive performance, with the highest AUROC (0.735), F-beta score (0.266), and specificity (0.970), but the lowest binary Brier score (0.142) (**Table 3**). For WHO growth standards, XGBoost performed best, yielding the highest AUROC (0.736), F-beta score (0.268), and specificity (0.968), with the lowest binary Brier score (0.142). Based on consistent multi-metric evaluation, the neural network (Chinese growth standards) and XGBoost (WHO growth standards) were selected as the best-performing models.

**Figure 2:**
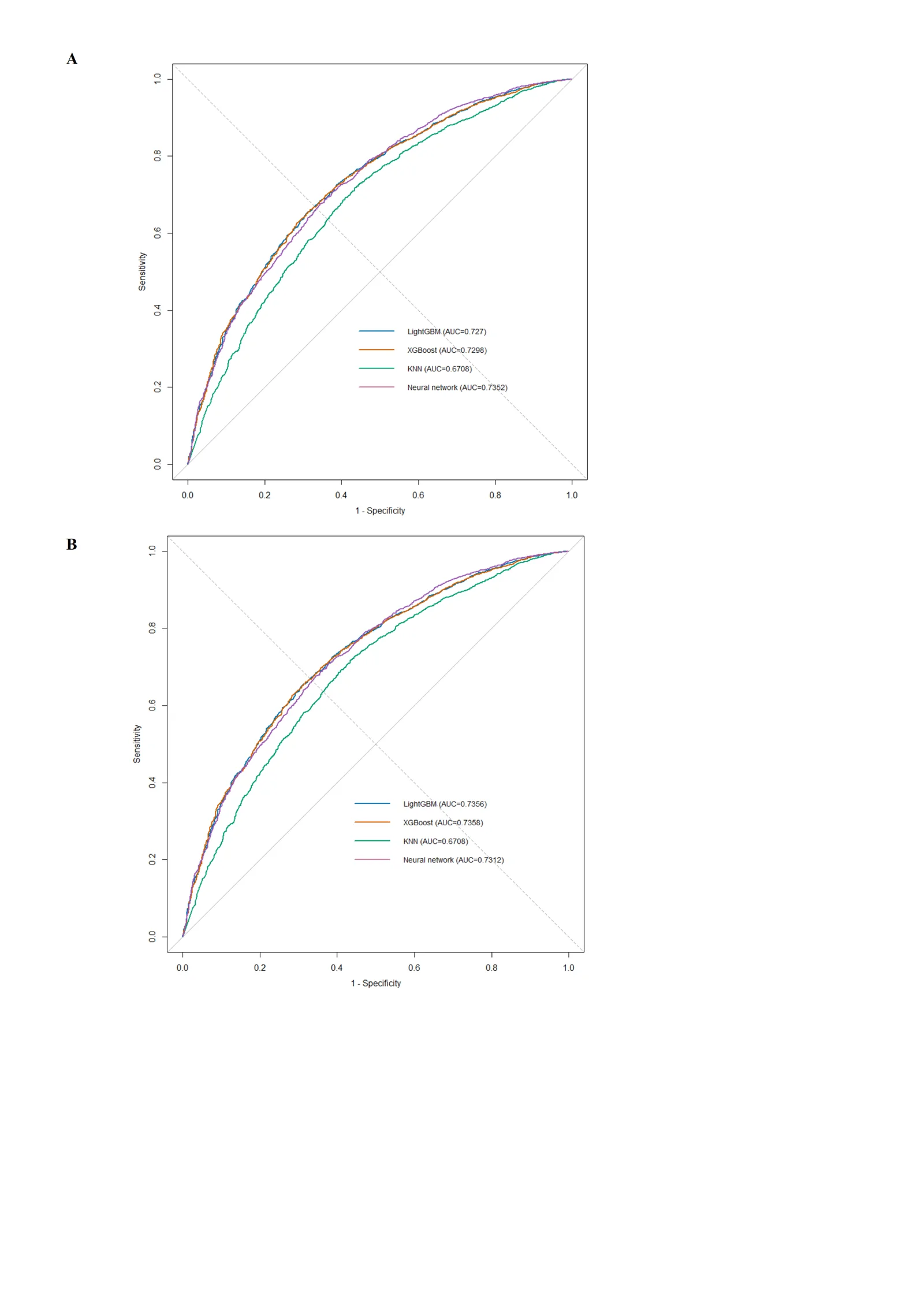
AUROCs of four machine learning models to predict obesity in children and adolescents. **Panel A.** Chinese growth standard. **Panel B.** WHO growth standard. AUROC – area under the receiver operating characteristic curve, LightGBM – light gradient boosting machine, NNET – neural network, XGBoost – eXtreme gradient boosting, WHO – World Health Organization.

**Table 2:** Performance comparison of four ML models for predicting obesity in children and adolescents*

	Chinese growth standards				WHO growth standards			
	**KNN**	**LightGBM**	**Neural network**	**XGBoost**	**KNN**	**LightGBM**	**Neural network**	**XGBoost**
**Accuracy**	0.795	0.789	0.806	0.791	0.795	0.805	0.804	0.806
**AUROC**	0.670	0.727	0.735	0.729	0.670	0.735	0.731	0.736
**Balanced accuracy**	0.544	0.544	0.570	0.545	0.544	0.557	0.557	0.571
**Binary Brier score**	0.154	0.151	0.142	0.150	0.154	0.142	0.143	0.142
**F*β*-Score**	0.192	0.183	0.266	0.181	0.195	0.226	0.227	0.268
**Precision**	0.492	0.623	0.591	0.661	0.492	0.600	0.581	0.584
**Sensitivity**	0.121	0.107	0.171	0.105	0.121	0.139	0.141	0.173
**Specificity**	0.967	0.981	0.970	0.984	0.967	0.976	0.974	0.968
**Calibration intercept**	−0.417	−0.324	−0.078	0.023	−0.625	−0.137	−0.163	0.121
**Calibration slope**	0.396	0.542	0.930	0.769	0.425	0.716	0.867	0.706

AUROC – area under the receiver operating characteristic curve, KNN – K-Nearest Neighbours, LightGBM – light gradient boosting machine, ML – machine learning, XGBoost – eXtreme gradient boosting, WHO – World Health Organization

*Model performance for binary classification (obesity *vs.* normal weight) was evaluated using core clinically meaningful metrics: discrimination (AUROC, area under the precision-recall curve, sensitivity and specificity) and calibration and overall error (Brier score, calibration intercept, and calibration slope).

**Table 3:** Optimal hyperparameters of four ML models for obesity prediction in children and adolescents

	Optimal hyperparameters
**Chinese growth criteria**	
KNN	K with 20
LightGBM	Learning rate with 0.12; bagging fraction 0.3; max_depth 1; num_threads 1; verbose −1
Neural network	MaxNWts 10,000; trace FALSE; decay 0.0445; maxit 167; size 5
XGBoost	Nrounds 1,000; nthread 1; verbose 0; eta 0.01; max_depth 2; subsample 0.8
**WHO growth criteria**	
KNN	K with 20
LightGBM	Learning rate with 0.56; bagging fraction 0.9; max_depth 1; num_threads 1; verbose −1
Neural network	MaxNWts 10,000; trace FALSE; decay 0.0667; maxit 200; size 3
XGBoost	Nrounds 1,000; nthread 1; verbose 0; eta 0.01; max_depth 5; subsample 0.5

KNN – K-nearest neighbour, LightGBM – light gradient boosting machine, ML – machine learning, XGBoost – eXtreme gradient boosting, WHO – World Health Organization

### Model interpretation

We applied SHAP analysis to the optimal feature subsets and best-performing models to improve interpretability, with higher SHAP values indicating stronger predictive contributions (**Figure 3**). Per Chinese growth standards, age was the most important feature, followed by maternal BMI, paternal BMI, screen time, and birthweight. Per WHO growth criteria, age remained the top predictor, followed by maternal BMI, paternal BMI, screen time, birthweight, paternal age at conception, gestational age, outdoor physical exercise duration, complementary feeding time, breastfeeding duration, birth length, and paternal weight. Bee-swarm plots confirmed that these features exhibited strong discriminatory power between participants with obesity and normal weight per both Chinese and WHO growth standards.

**Figure 3:**
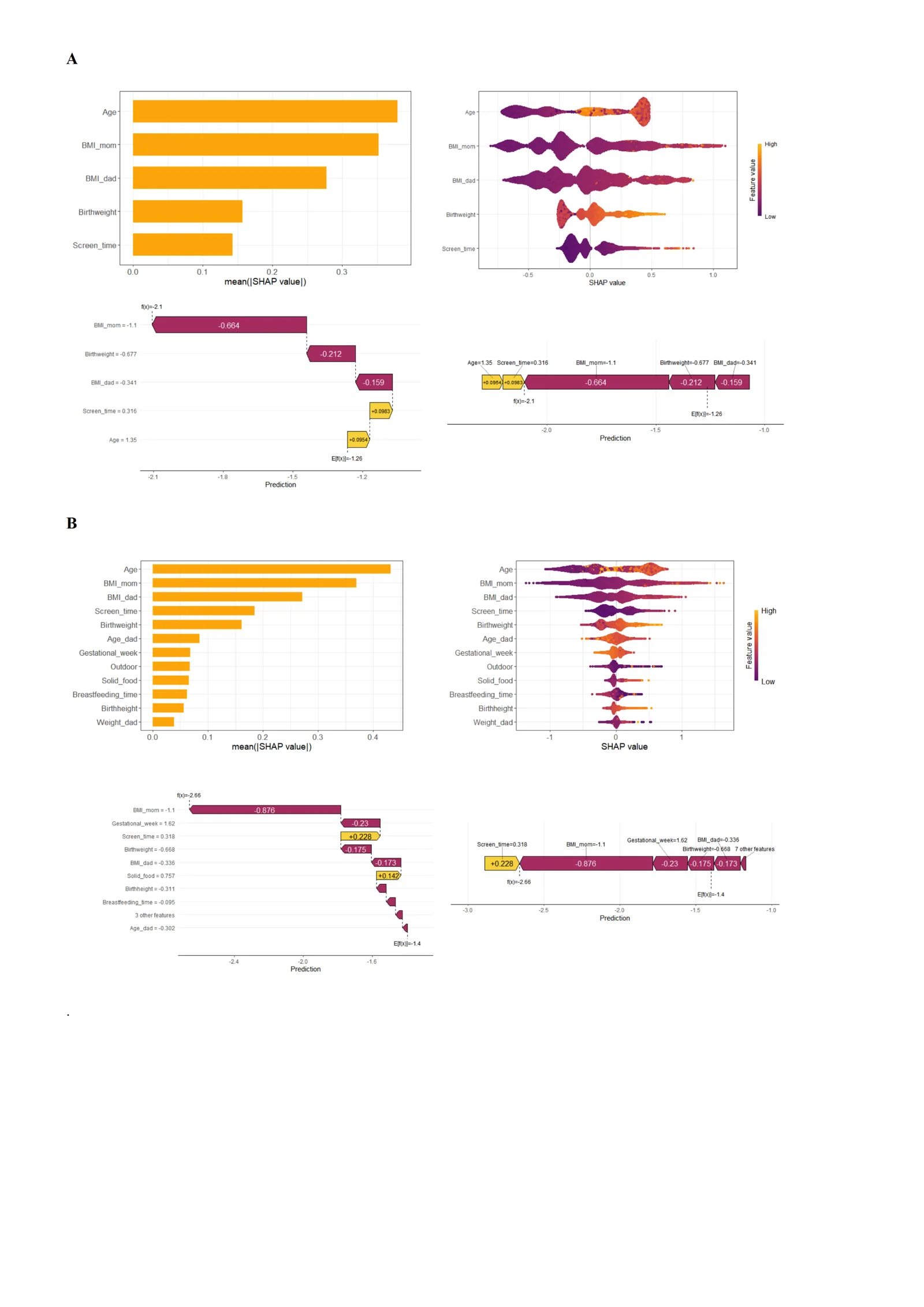
Global SHAP interpretation of key features to predict obesity in children and adolescents. **Panel A.** Chinese growth standard. **Panel B.** WHO growth standard. BMI – body mass index, SHAP – SHapley Additive exPlanations.

Per Chinese growth standards, maternal BMI, birthweight, and paternal BMI drove obesity risk, while screen time and age exerted protective or counterbalancing effects. Per WHO growth standards, maternal BMI, gestational age, birthweight, paternal BMI, breastfeeding duration, and paternal age at conception potentiated risk, whereas screen time and complementary feeding time were associated with reduced predicted risk.

Subgroup analyses confirmed robust model performance across the entire age range (Table S3 in the **Online Supplementary Document**), supporting stable predictive performance despite age-related heterogeneity in obesity classification.

Regarding calibration, which evaluates the agreement between predicted probabilities and observed outcomes, the neural network showed optimal global metrics under the Chinese standards (intercept = −0.078; slope = 0.930), whereas visual LOESS curves indicated that XGBoost and LightGBM provided the best local calibration under the Chinese and WHO standards, respectively (Figure S2 in the **Online Supplementary Document**). Exploratory post-hoc recalibration *via* Platt scaling yielded negligible statistical improvements, and thus native probabilities were evaluated without post-hoc adjustments to reflect their unaltered practical performance in facilitating risk-based screening.

### External validation

We externally validated the final models in an independent cohort from Tangshan, Hebei province (n = 5,343). Per both the Chinese growth criteria (AUROC = 0.699) and WHO growth criteria (AUROC = 0.707), the model achieved satisfactory results (Figure S1 in the **Online Supplementary Document**). These results indicated satisfactory generalisability and transportability across geographic regions and sociodemographic backgrounds, despite differences in socioeconomic and school-related characteristics between Beijing and Hebei.

### Model deployment

To support clinical and public health translation, we deployed the best-performing models with minimal feature sets as interactive web-based applications for obesity risk in children and adolescents per Chinese [35] and WHO [36] growth standards (Figure S2 in the **Online Supplementary Document**).

These applications serve two primary user groups: primary care clinicians for auxiliary screening aid, and parents/caregivers for early obesity risk assessment. To ensure ethical use and clinical safety, clear disclaimers are displayed, emphasising that the tools provide probabilistic risk stratification only and do not replace formal clinical diagnosis.

Regarding data privacy and security, all user inputs are processed entirely on the client side. No personally identifiable information, input data, or IP addresses are transmitted to or stored on external servers. The platform functions as an anonymous, stateless calculator with no user tracking or data retention. This architecture fully protects user privacy and meets ethical standards for data handling and informed consent in minor populations.

## DISCUSSION

We investigated associations between multi-domain factors and obesity risk among children and adolescents aged 3−18 years per both Chinese and WHO growth standards. Of 37 eligible features, we retained 5 for the Chinese and 12 for WHO growth standards, identifying two best-performing ML models per Chinese (neural network) and WHO (XGBoost) growth standards. We visually interpreted model predictions using global and local SHAP analyses, and deployed the best-performing models as online prediction tools for real-world application. To our knowledge, this is the first ML study to internally and externally develop predictive models for paediatric obesity per both Chinese and WHO growth standards.

Obesity in children and adolescents is rising globally, with varying prevalence estimates often reflecting divergent definitional criteria. To improve the generalisability of our findings, we defined obesity using both Chinese and WHO growth standards. Under both definitions, LASSO regression, which effectively handles high-dimensional data by shrinking irrelevant coefficients while retaining informative features, consistently identified five shared features: child age, maternal BMI, paternal BMI, screen time, and birthweight. Obesity has also been described as a socially contagious epidemic [37]. The influence of family environment, including parental dietary modelling, on children’s eating behaviours supports the view proposed by Cohen-Cole and Fletcher [38] that the spread of obesity is closely tied to shared living. In addition, Christakis and Fowler analysed a large social network of 12,067 participants from the Framingham Heart Study followed from 1971 to 2003 and reported that obesity appeared to spread *via* social connections [39]. Thus, targeting a single domain of risk factors is insufficient to capture the complex and multifactorial aetiology of childhood obesity.

To provide further insight, we collected data covering five domains informed by existing obesity literature: demographic characteristics, fetal and early-life factors, lifestyle behaviours, health status, and family information. In total, we included 37 features with missingness rates <30%, a threshold commonly used in data-driven research to balance variable retention and data robustness. Many features exhibited complex interrelationships: parental BMI and children’s dietary patterns were strongly correlated, yet socioeconomic status and physical activity formed interdependent networks. Such multicollinearity is inherent to real-world epidemiological data and reflects the multifactorial nature of childhood obesity. Traditional statistical approaches often assume variable independence, an assumption frequently violated under multicollinearity, leading to unstable estimates and inflated type I error. This limitation supports the use of flexible analytical frameworks that can accommodate complex feature structures. We therefore adopted four widely used ML algorithms to account for multicollinearity, generate reliable risk estimates, and enable visualisation of obesity risk.

Following systematic hyperparameter tuning and performance comparison, we selected the two best-performing models to predict obesity in children and adolescents using both Chinese (neural network) and WHO (XGBoost) growth standards. Neural networks, core components of modern artificial intelligence, are brain-inspired computational systems that simulate interconnected neuronal activity to learn patterns, process information, and support decision-making. This approach has been increasingly applied in obesity research. For example, Huang *et al.* used deep neural networks to predict obesity from short audio recordings, achieving a global accuracy of 0.70 and a macro F1-score of 0.65 [40]. Leo *et al.* developed a lightweight convolutional neural network for early obesity identification using thermal images, reporting an accuracy of 83.08% [41]. In this study, the neural network achieved 80.68% accuracy for obesity per Chinese growth standards, outperforming the other three models. Additionally, XGBoost is a highly efficient gradient-boosting algorithm with strong performance in ML challenges and real-world applications, and has also been widely adopted in obesity-related research. For instance, Wu *et al.* used the top 40 bacterial taxa as inputs to four ML models for obesity prediction and reported that XGBoost achieved the highest accuracy (81.44%) [42]. In our study, XGBoost yielded the highest accuracy (80.63%) for obesity per WHO growth standards. We also reported detailed hyperparameter tuning procedures for all models, a critical step given evidence that hyperparameter optimisation can improve XGBoost AUROC by up to 5% [33]. We optimised hyperparameters *via* grid search and cross-validation to improve computational efficiency and ensure stable results.

Although our models achieved acceptable discrimination (AUROC ~ 0.73), the default probability threshold yielded relatively low sensitivity but high specificity. Our web-based tools should therefore be viewed as high-specificity risk-stratification instruments rather than general population screening tools. This conservative threshold minimises false positives, allowing clinicians and public health practitioners to reliably prioritise high-risk children and adolescents for intensive targeted interventions.

We used further SHAP analysis to address the ‘black-box’ nature of ML and improve the interpretability of obesity prediction. Both global and local SHAP values confirmed the discriminative utility of the selected features. To translate findings into practice, we developed a publicly available online application that predicts paediatric obesity risk per both Chinese and WHO growth standards and provides visual explanations of each feature’s contribution. This tool supports real-world use by parents, researchers, and clinicians.

### Limitations

Several limitations warrant consideration. First, the cross-sectional design precludes causal inference between identified risk factors and obesity. Second, participants were recruited from a single district in Beijing, which may restrict generalisability to more diverse populations. Third, except for measured height and weight, all data were collected *via* electronic questionnaires; recall bias cannot be fully excluded. Key predictors such as parental BMI and screen time were parent-reported, which may introduce measurement error and social desirability bias. Objective validation of self-reported variables was not feasible due to the large sample size and strict anonymity protocols. Nevertheless, such biases likely had minimal influence on feature importance rankings, and the models’ satisfactory predictive performance suggests robustness to real-world data noise. Fourth, we did not include overweight in the outcome definition. While this choice was intentional to identify robust predictors of a severe phenotype, it may introduce spectrum bias. Our web-based tool functions as an early warning system for clinical obesity rather than a continuous BMI monitoring instrument, and thresholds optimised for this extreme contrast may yield ambiguous risk scores for overweight individuals. Lastly, although we assessed five domains of obesity-related factors, we did not include other important determinants, such as broader social and environmental exposures.

## CONCLUSIONS

We identified two interpretable ML models for obesity in children and adolescents per both Chinese and WHO growth standards, each demonstrating modest but reliable predictive performance. To facilitate translation into practice, we developed a user-friendly online prediction tool. This study represents an important step toward actionable risk stratification for paediatric obesity, with its full impact dependent on iterative refinement, external validation, and interdisciplinary collaboration between researchers, clinicians, and public health professionals.

## Additional material


Online Supplementary Document

## Data Availability

**Data availability:** Because the dataset includes identifiable minor information and private data, the full dataset cannot be publicly shared. Requests for access to the de-identified data should be directed to the corresponding authors.
