# Supplementary material for: External validation and application of machine learning models for obesity in Chinese children and adolescents per Chinese and WHO growth standards
Source: J Glob Health. 2026 Oct 19;16:04299. doi: 10.7189/jogh.16.04299 (PMC13679347; doi:10.7189/jogh.16.04299)

Supplement to: Zheng F, Wang X, Wang Q, Zhang W, Zhang Z, Niu W. External validation and application of machine learning models for obesity in Chinese children and adolescents per Chinese and WHO growth standards. J Glob Health. 2026;16:04299.

Table S1. Performance comparison of five machine learning models for predicting childhood and adolescent obesity assessed by both Chinese and WHO standards.
Table S2.Detailed item wording, response options, recall periods, and numeric coding rules of the self-designed questionnaire.
Table S3.Subgroup analysis of the optimal predictive model across the 3–6, 7–12, and 13–18 year age groups.
Appendix S1. Survey Questionnaire on Growth, Development, and Behavioral Risk Factors of Childhood Obesity.
Figure S1.Receiver operating characteristic (ROC) curves of the final predictive models in the external validation cohort.
Figure S2.Calibration curves of the four machine learning models in the validation set.
Figure S3.LASSO coefficient path diagram screening features and regression cross-validation curves. Panel A. Chinese growth standard. Panel B. WHO growth standard. LASSO – Least Absolute Shrinkage and Selection Operator, WHO – World Health Organization
Figure S4. Online application tool for the prediction of childhood and adolescent obesity assessed by Chinese (Panel A) and WHO (Panel B) standards.

Table S1. Performance comparison of five machine learning models for predicting childhood and adolescent obesity assessed by both Chinese and WHO standards.

Metric	Chinese standards for obesity definition				WHO standards for obesity definition			
	**KNN**	**LightGBM**	**Neural network**	**XGBoost**	**KNN**	**LightGBM**	**Neural network**	**XGBoost**
Accuracy	0.7953	0.7898	0.8068	0.7917	0.7953	0.8056	0.8041	0.8063
Area Under the ROC Curve	0.6708	0.7270	0.7352	0.7298	0.6708	0.7356	0.7312	0.7358
Balanced Accuracy	0.5448	0.5446	0.5705	0.5450	0.5448	0.5579	0.5576	0.5711
Binary Brier Score	0.1548	0.1512	0.1423	0.1504	0.1548	0.1424	0.1431	0.1423
Classification Error	0.2047	0.2102	0.1932	0.2083	0.2047	0.1944	0.1959	0.1937
Diagnostic Odds Ratio	4.1803	6.4686	6.6113	7.6492	4.1803	6.6626	6.1586	6.4374
Fβ-Score	0.1953	0.1833	0.2656	0.1813	0.1953	0.2264	0.2273	0.2680
False Discovery Rate	0.5071	0.3767	0.4085	0.3384	0.5071	0.3992	0.4182	0.4154
False Negatives	995	1113	939	1116	995	975	973	936
False Negative Rate	0.8782	0.8925	0.8288	0.8949	0.8782	0.8605	0.8588	0.8261
False Omission Rate	0.1886	0.2037	0.1796	0.2036	0.1886	0.1842	0.1843	0.1794
False Positives	142	81	134	67	142	105	115	140
False Positive Rate	0.0321	0.0183	0.0303	0.0151	0.0321	0.0237	0.0260	0.0317
Logarithmic Loss	0.6255	0.4691	0.4467	0.4673	0.6255	0.4462	0.4487	0.4465
Multiclass AUC Type 1 Pairwise	0.6708	0.7270	0.7352	0.7298	0.6708	0.7356	0.7312	0.7358
Multiclass AUC Type 1 Unweighted	0.6708	0.7270	0.7352	0.7298	0.6708	0.7356	0.7312	0.7358
Multiclass AUC Type N Pairwise	0.6708	0.7270	0.7352	0.7298	0.6708	0.7356	0.7312	0.7358
Multiclass AUC Type N Unweighted	0.6708	0.7270	0.7352	0.7298	0.6708	0.7356	0.7312	0.7358

Multiclass AUC Macro-Averaged	0.6708	0.7270	0.7352	0.7298	0.6708	0.7356	0.7312	0.7358
Multiclass Brier Score	0.3096	0.3023	0.2847	0.3008	0.3096	0.2848	0.2863	0.2846
Matthews Correlation Coefficient	0.1652	0.1934	0.2409	0.2030	0.1652	0.2195	0.2140	0.2401
Negative Predictive Value	0.8114	0.7963	0.8204	0.7964	0.8114	0.8158	0.8157	0.8206
Positive Predictive Value	0.4929	0.6233	0.5915	0.6616	0.4929	0.6008	0.5818	0.5846
Precision-Recall AUC	0.3481	0.4337	0.4212	0.4445	0.3481	0.4218	0.4170	0.4226
Precision	0.4929	0.6233	0.5915	0.6616	0.4929	0.6008	0.5818	0.5846
Sensitivity	0.1218	0.1075	0.1712	0.1051	0.1218	0.1395	0.1412	0.1739
Specificity	0.9679	0.9817	0.9697	0.9849	0.9679	0.9763	0.9740	0.9683
True Negatives	4280	4352	4288	4366	4280	4317	4307	4282
True Negative Rate	0.9679	0.9817	0.9697	0.9849	0.9679	0.9763	0.9740	0.9683
True Positives	138	134	194	131	138	158	160	197
True Positive Rate	0.1218	0.1075	0.1712	0.1051	0.1218	0.1395	0.1412	0.1739
Calibration Intercept	-0.417	-0.324	-0.078	0.023	-0.625	-0.137	-0.163	0.121
Calibration Slope	0.396	0.542	0.930	0.769	0.425	0.716	0.867	0.706

Abbreviations: AUC, area under the ROC; KNN, K-Nearest Neighbors; LightGBM, light gradient boosting machine; ROC, receiver operating characteristic; XGBoost, eXtreme gradient boosting; WHO, World Health Organization.

Table S2.Detailed item wording, response options, recall periods, and numeric coding rules of the self-designed questionnaire.

Domain	Variable	Questionnaire Item / Variable Definition	Recall Period	Categorization / Coding Rule
**Demographic Characteristics**	Age	What is the child's date of birth? (Used to calculate age in months up to the survey date).	Not applicable	Continuous variable (accurate to 1 month)
	Sex	What is the child's sex?	Not applicable	Categorical: Male; Female
	Height	Standardized measurement conducted by health practitioners in kindergartens or schools.	At the time of survey	Continuous variable (accurate to 0.01 cm)
	Weight	Standardized measurement conducted by health practitioners in kindergartens or schools.	At the time of survey	Continuous variable (accurate to 0.01 kg)
	Body Mass Index (BMI) & BMI z-score	Calculated as weight (kg) divided by height squared (m^2). Overweight and obesity are defined according to the WHO Child Growth Standards (2006) for children aged 0-5 years and the WHO Growth Reference (2007) for ages 5-19.	At the time of survey	Continuous variable (accurate to 0.01 kg/m^2). Categorical thinness: BMI-z < -2 SD; Overweight: +1 SD < BMI-z <= +2 SD; Obesity: BMI-z > +2 SD.
**Early Life Factors**	Gestational age	What was the child's gestational age at birth?	At birth	Categorized based on WHO criteria: Preterm (<37 weeks); Term (37 to <42 weeks); Post-term (>=42 weeks). Continuous values accurate to 1 week.
	Delivery mode	What was the mode of delivery?	At birth	Categorical: Vaginal delivery; Cesarean section; Forceps delivery; Other
	Gravidity (Pregnancy order)	Which pregnancy was this for the mother (including induced abortions, spontaneous abortions, and normal pregnancies)?	Lifetime (up to child's birth)	Continuous / Integer
	Parity (Birth order)	What is the birth order of this child for the mother?	Lifetime (up to child's birth)	Continuous / Integer
	Twin or multiple birth	Is the child a twin or part of a multiple birth?	At birth	Categorical: Yes; No
	Birth length	What was the child's length at birth?	At birth	Continuous variable (accurate to 0.1 cm)
	Birth weight	What was the child's weight at birth?	At birth	Continuous variable (accurate to 1 g)
	Infant feeding practice	What was the primary feeding practice during the child's first 6 months of life?	First 6 months of life	Categorical: Exclusive breastfeeding; Partial breastfeeding (mixed breast milk and formula); Non-breastfeeding (formula only)

Domain	Variable	Questionnaire Item / Variable Definition	Recall Period	Categorization / Coding Rule
	Duration of breastfeeding	If answered 'exclusive' or 'partial' breastfeeding: at what age (in months) was the child completely weaned off breast milk?	Infancy	Continuous variable (accurate to 1 month)
	Timing of complementary feeding	At what age (in months) were complementary foods introduced to the child?	Infancy	Continuous variable (accurate to 1 month)
**Lifestyle Factors**	Sedentary time	Asked separately for weekdays and weekends: On average, how much time per day does the child spend sedentary (including attending classes, watching TV, reading, studying, doing homework, etc.)?	Typical day (recent past)	Continuous variable (accurate to 0.5 hours). Calculated as: [(Weekday avg * 5) + (Weekend avg * 2)] / 7
	Screen time	Asked separately for weekdays and weekends: On average, how much time per day does the child use electronic devices for learning (e.g., online classes) and for entertainment (e.g., TV, mobile phones)?	Typical day (recent past)	Continuous variable (accurate to 0.5 hours). Calculated as: [(Weekday avg * 5) + (Weekend avg * 2)] / 7. (Total = Learning time + Entertainment time)
	Outdoor exercise time	Asked separately for weekdays and weekends: On average, how much time per day does the child spend on outdoor physical activities (e.g., running, ball games, cycling, walking)?	Typical day (recent past)	Continuous variable (accurate to 1 minute). Calculated as: [(Weekday avg * 5) + (Weekend avg * 2)] / 7
	Bedtime	What time does the child usually go to bed at night?	Typical night (recent past)	Continuous variable (accurate to 1 hour)
	Eating speed	On average, how much time does the child take to finish a meal?	Typical meal (recent past)	Categorical: (1) <15 minutes; (2) 15–30 minutes; (3) >=30 minutes
	Frequency of consuming Western fast food	How often does the child eat Western fast food or fried foods (e.g., French fries, fried chicken, pizza)?	Typical week (recent past)	Categorical: None or occasionally; 1-2 times/week; 3-5 times/week; Almost daily
	Frequency of consuming sweets	How often does the child eat sweets (e.g., candies, cakes, chocolates, desserts, sugar-sweetened beverages)?	Typical week (recent past)	Categorical: None or occasionally; 1-2 times/week; 3-5 times/week; Almost daily
	Frequency of consuming late-night	How often does the child eat late-night snacks (defined as eating between 22:00 and 04:00)?	Typical week (recent past)	Categorical: None or occasionally; 1-2 times/week; 3-5 times/week; Almost daily

Domain	Variable	Questionnaire Item / Variable Definition	Recall Period	Categorization / Coding Rule
	snacks			
**Health Status**	Food allergy	Does the child have a history of food allergies (e.g., developing skin redness, swelling, rash, abdominal pain, etc., within 2 hours after consuming certain foods)?	Lifetime	Categorical: Yes; No
	Drug allergy	Is the child allergic to any medications, or have they experienced allergic symptoms (e.g., skin itching or rash) after taking a medication?	Lifetime	Categorical: Yes; No
**Family Information**	Maternal childbearing age	Calculated by subtracting the child's current age from the mother's current age.	At the time of survey	Continuous variable (accurate to 1 year)
	Paternal childbearing age	Calculated by subtracting the child's current age from the father's current age.	At the time of survey	Continuous variable (accurate to 1 year)
	Maternal BMI	Calculated using the mother's self-reported height and weight (Weight in kg / Height in m^2).	At the time of survey	Continuous variable (accurate to 0.01 kg/m^2)
	Paternal BMI	Calculated using the father's self-reported height and weight (Weight in kg / Height in m^2).	At the time of survey	Continuous variable (accurate to 0.01 kg/m^2)
	Maternal education level	What is the highest level of education completed by the mother?	At the time of survey	Categorical: High school or below; Bachelor's degree; Master's degree or above
	Paternal education level	What is the highest level of education completed by the father?	At the time of survey	Categorical: High school or below; Bachelor's degree; Master's degree or above
	Annual household income	What is the approximate total annual income of the household?	Past year	Categorical: <100,000 RMB; 100,000–300,000 RMB; >=300,000 RMB
	Family history of diabetes	Have any immediate family members of the child (including parents, grandparents, siblings) been diagnosed with diabetes? If yes, specify the number of affected relatives.	Lifetime (Family history)	Categorical: Yes; No. (If yes, record the number of cases)
	Family history of hypertension	Have any immediate family members of the child (including parents, grandparents, siblings) been diagnosed with hypertension? If yes, specify the number of affected relatives.	Lifetime (Family history)	Categorical: Yes; No. (If yes, record the number of cases)

Table S3.Subgroup analysis of the optimal predictive model across the 3–6, 7–12, and 13–18 year age groups.

Age Group	Overall	3–6 years	7–12 years	13–18 years
Optimal Model (Chinese Standard: XGBoost) AUROC (95% CI)	0.7352	0.6825	0.7801	0.6644
Optimal Model (WHO Standard: LightGBM) AUROC (95% CI)	0.7358	0.6643	0.7459	0.6919

Abbreviations: AUC, area under the ROC;

Appendix S1. Survey Questionnaire on Growth, Development, and Behavioral Risk Factors of Childhood Obesity.

Dear Parents:

Questionnaire on Children's Growth, Development, and Risk Factors

Hello! Children are the hope of our families and the future of our nation. Children's health, particularly their weight status, has always been a focus of societal concern. Currently, childhood underweight, overweight, and obesity have become global public health challenges. Poor weight status not only affects current health but also has profound negative impacts on the future. For example, being underweight may lead to weakened immunity, stunted growth, and impaired intellectual development; whereas overweight and obesity increase the risk of chronic diseases such as cardiovascular disease, diabetes, non-alcoholic fatty liver disease, and precocious puberty.

Therefore, this study has developed this questionnaire to deeply understand the current prevalence of childhood underweight, overweight, and obesity, and to explore the underlying influencing factors. This will provide references for the prevention of susceptible school-aged children and guide weight reduction for obese children.

Important Notices Before Filling Out the Questionnaire:

Your participation in this questionnaire is completely voluntary, and you may withdraw at any time.

The information collected will solely be used to aggregate the incidence and risk factors of childhood obesity in the entire region. The personal information of the children involved will be kept strictly confidential.

Filling Instructions:

This questionnaire system will evaluate and provide feedback on your child's growth, development, and mental health based on the filled content. It is recommended that the child's mother fill out this questionnaire to ensure the accuracy of the content and improve the validity of the evaluation results.

During the answering process, parents only need to focus on the text following the "=" sign. The numbers preceding the "=" are required for the questionnaire format

and are irrelevant to the content.

For numerical information required in the questionnaire, please pay attention to the units provided: for example, the standard unit for height is "centimeters" (cm), where 100 cm = 1 meter; the unit for weight is "kilograms" (kg) or "grams" (g), where 1 kg = 2 jin = 1000 g.

Your support and cooperation are of great significance to your child. Thank you again for your participation!

Part I: Basic Information

District/City ______ School ______ Grade/Class (Please provide full name) ______

Gender: ☐ 1=Male ☐ 2=Female

Date of Birth (Gregorian calendar): Year ___ Month ___ Day ___

Ethnicity: ______

Father's Date of Birth: ______; Mother's Date of Birth: ______

Father's Height: ______ cm; Mother's Height: ______ cm

Father's Weight: ______ kg; Mother's Weight: ______ kg

Father's Education Level:
☐ 1=Junior high school or below
☐ 2=High school/Vocational school

☐ 3=Bachelor's degree/Associate degree
☐ 4=Master's degree or above

Mother's Education Level:
☐ 1=Junior high school or below
☐ 2=High school/Vocational school
☐ 3=Bachelor's degree/Associate degree
☐ 4=Master's degree or above

Annual Household Income:
☐ 1=Under 100,000 RMB
☐ 2=100,000 to 300,000 RMB
☐ 3=300,000 RMB and above

Part II: Early Life Factors

This child is the mother's ___th pregnancy (including induced abortions, spontaneous abortions, and normal pregnancies), and the mother's ___th birth.
(Example: If the child has an older brother, and the mother had one miscarriage before giving birth to this child, then this child is the 3rd pregnancy and the 2nd birth.)

Child's Gestational Age: ___ weeks

Child's Birth Weight: ___ grams; Child's Birth Length: ___ cm

Child's Mode of Delivery: ☐ Vaginal delivery ☐ Cesarean section ☐ Forceps delivery ☐ Other
4.1 If Cesarean section, the reason was:

☐ Abnormal fetal position (e.g., transverse lie, footling presentation)
☐ Twin or multiple pregnancy
☐ Large fetal size
☐ Maternal condition unsuitable for vaginal delivery (e.g., birth canal abnormality, genital tract infection, pregnancy with tumor, heart disease, respiratory disease, eclampsia, etc.)
☐ Emergency situation requiring immediate fetal rescue (e.g., fetal distress, umbilical cord prolapse, placental abruption, etc.)
☐ Other (e.g., voluntary Cesarean section)

Is your child a twin or part of a multiple birth?
☐ 1=No ☐ 2=Yes

Feeding method during the child's first 6 months:
☐ Exclusive breastfeeding
☐ Mixed feeding (Breast milk + Formula)
☐ Artificial feeding (Formula only)
6.1 At what age (in months) was the child completely weaned off breast milk? ___ months

At what age (in months) were complementary foods introduced to the child? ___ months

Part III: Children's Lifestyle Assessment

How much time does your child usually spend on outdoor physical activities (e.g., running on the playground, playing ball, cycling, mountain climbing)?
Workdays: ___ hours/day; Rest days (Weekends/Holidays): ___ hours/day

What is your child's total usual sedentary time (including attending classes, watching TV, reading, studying, doing homework, etc.)?
Workdays: ___ hours/day; Rest days: ___ hours/day

On average, how much time per day does your child spend using electronic devices for learning (including online classes, completing homework, etc.)?
Workdays: ___ hours/day; Rest days: ___ hours/day

On average, how much time per day does your child spend using electronic devices for entertainment (time spent on TVs, mobile phones, tablets, or going to the movies, not for work or study purposes)?
Workdays: ___ hours/day; Rest days: ___ hours/day

What time does your child usually go to bed at night? (Please use the 12-hour clock)
Workdays: ___ PM; Rest days: ___ PM

Your child usually takes ___ minutes for breakfast, ___ minutes for lunch, and ___ minutes for dinner.
(Choose one: ☐ 1=Less than 15 minutes ☐ 2=15-30 minutes ☐ 3=30 minutes or more)

How often does your child eat Western fast food or fried foods (e.g., French fries, fried chicken, pizza)?
☐ 1=None or occasionally ☐ 2=1-2 times/week ☐ 3=3-5 times/week ☐ 4=Almost daily

How often does your child eat sweets (e.g., sugar-sweetened beverages, candies, cakes, chocolates)?
☐ 1=None or occasionally ☐ 2=1-2 times/week ☐ 3=3-5 times/week ☐ 4=Almost daily

How often does your child eat late-night snacks?
☐ 1=None or occasionally ☐ 2=1-2 times/week ☐ 3=3-5 times/week ☐ 4=Almost daily

Part IV: Personal and Family Health Status

Has your child currently or ever been diagnosed with the following chronic diseases?

☐ 1=None ☐ 2=Chronic kidney disease ☐ 3=Congenital heart disease ☐ 4=Other (Please specify the disease name: _______)

Do any of the following immediate family members of the child have hypertension? (Requires diagnosis from a tertiary Grade A hospital, multiple choices allowed)
☐ 1=None ☐ 2=Father ☐ 3=Mother ☐ 4=Maternal Grandmother ☐ 5=Maternal Grandfather ☐ 6=Paternal Grandfather ☐ 7=Paternal Grandmother ☐ 8=Siblings

Do any of the following immediate family members of the child have diabetes? (Requires diagnosis from a tertiary Grade A hospital, multiple choices allowed)
☐ 1=None ☐ 2=Father ☐ 3=Mother ☐ 4=Maternal Grandmother ☐ 5=Maternal Grandfather ☐ 6=Paternal Grandfather ☐ 7=Paternal Grandmother ☐ 8=Siblings

Does your child have a history of food allergies (e.g., experiencing skin redness, swelling, rash, abdominal pain, etc., within 2 hours after eating a certain food)?
☐ 1=No ☐ 2=Yes

Is your child known to be allergic to any medications? Or has your child experienced allergic symptoms such as skin itching or rash after taking any medication?
☐ 1=No ☐ 2=Yes

Figure S1.Receiver operating characteristic (ROC) curves of the final predictive models in the external validation cohort.

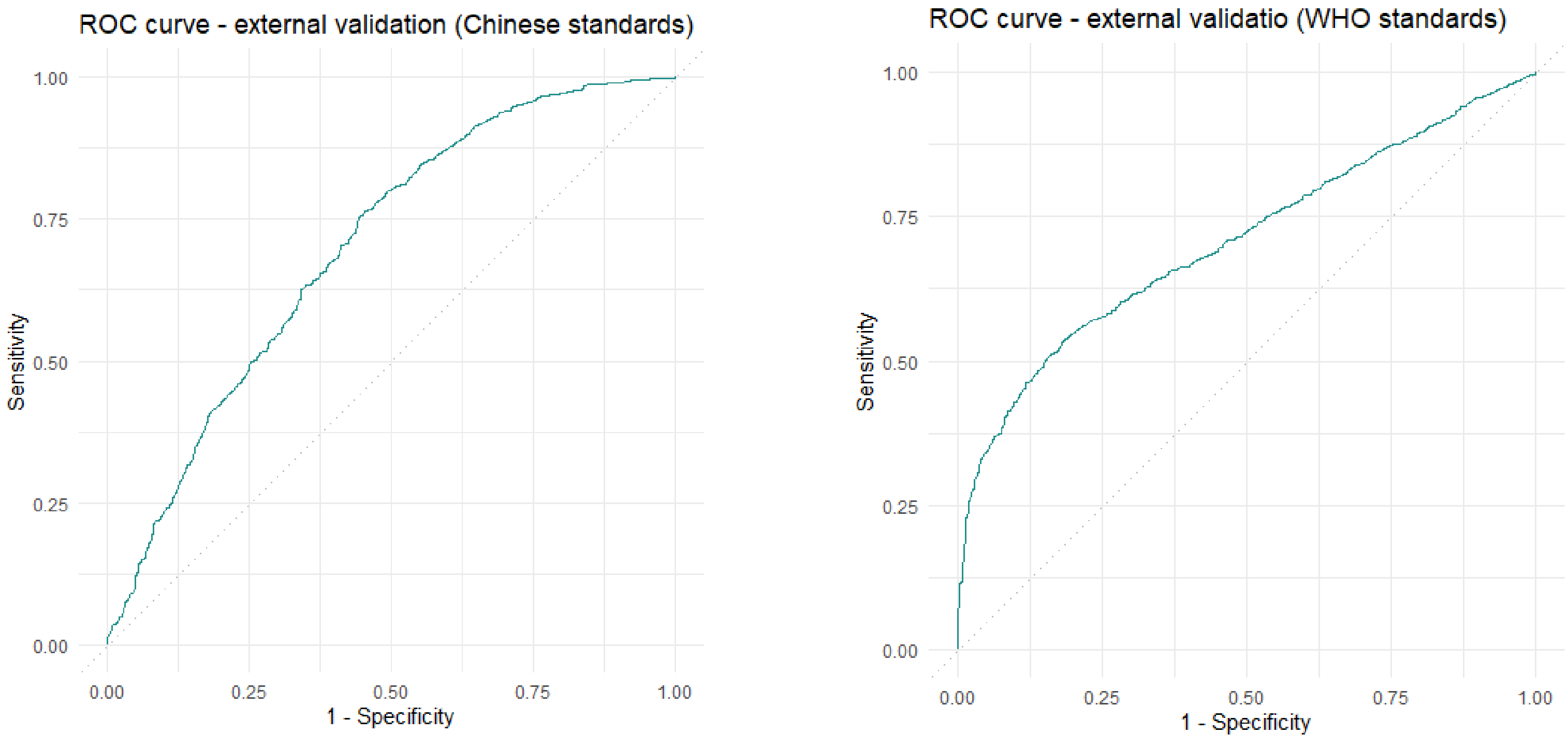

(A) (B)

(A) ROC curve based on the Chinese growth criteria, achieving an area under the ROC curve (AUROC) of 0.699. (B) ROC curve based on the World Health Organization (WHO) growth criteria, achieving an AUROC of 0.707. The external validation was conducted using an independent cohort from Tangshan city, Hebei province (n = 5343).

Figure S2.Calibration curves of the four machine learning models in the validation set.

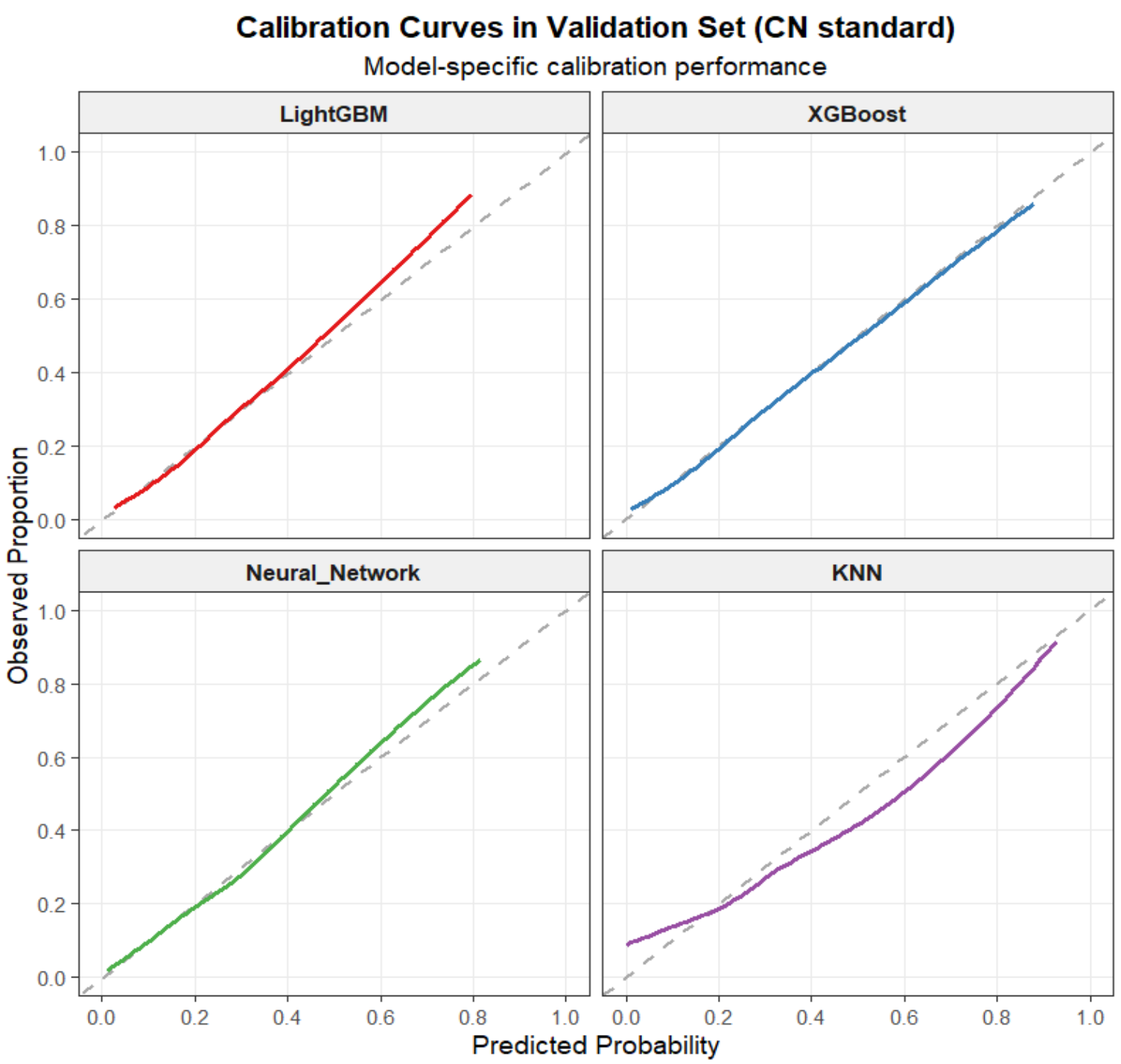

(A)

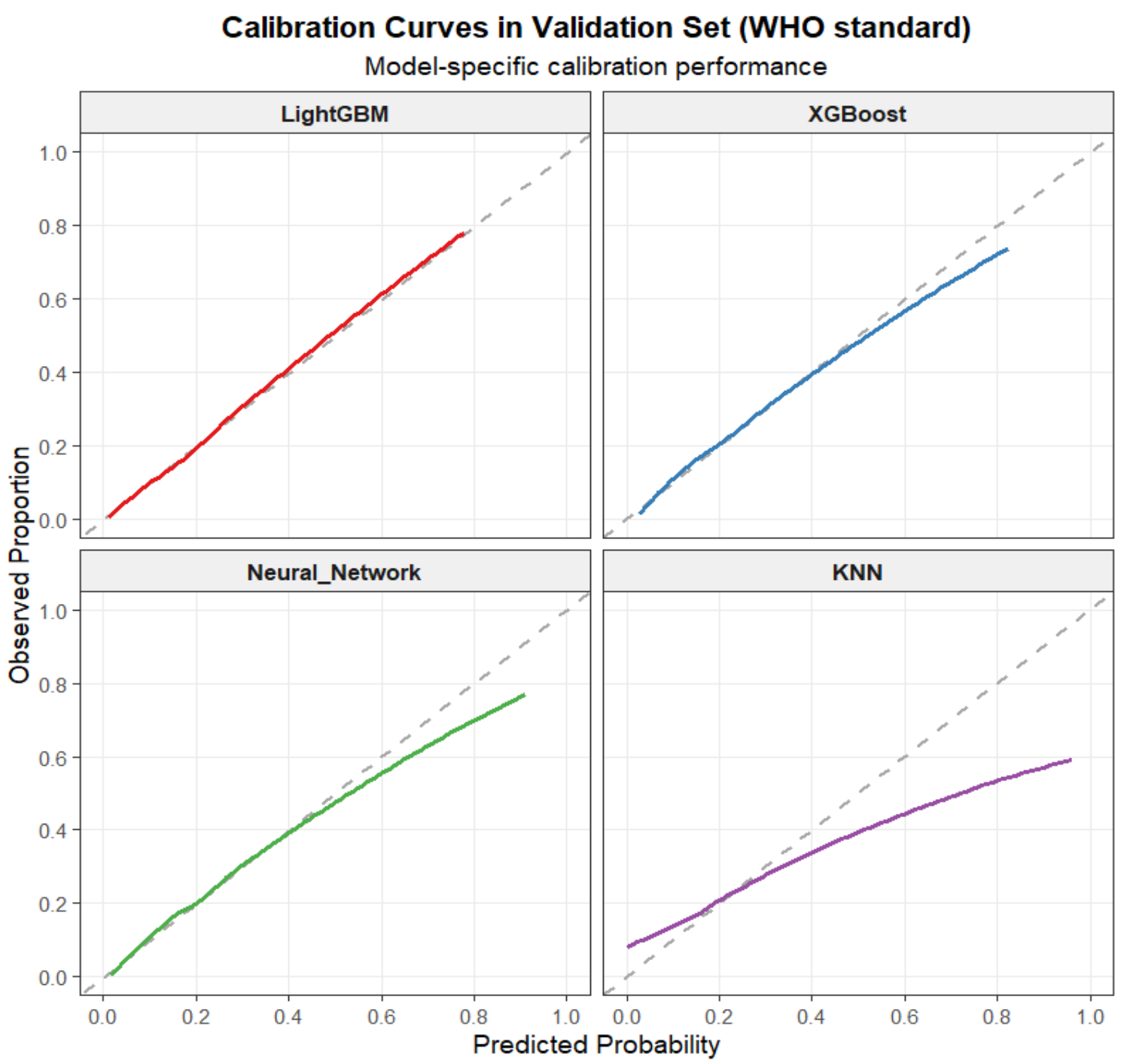

(B)

(A) Calibration curves based on the Chinese (CN) standard. (B) Calibration curves based on the World Health Organization (WHO) standard.

A

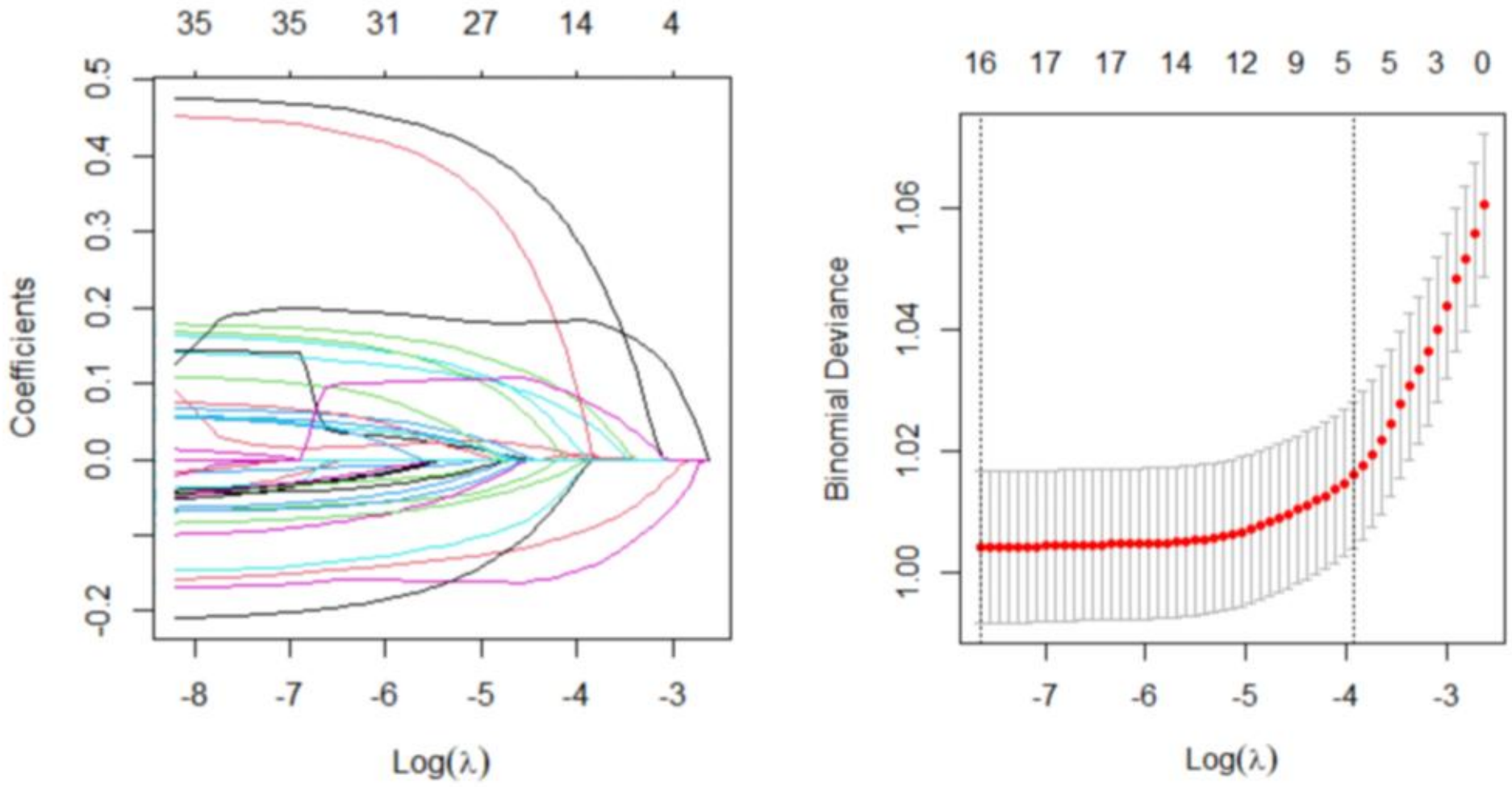

B

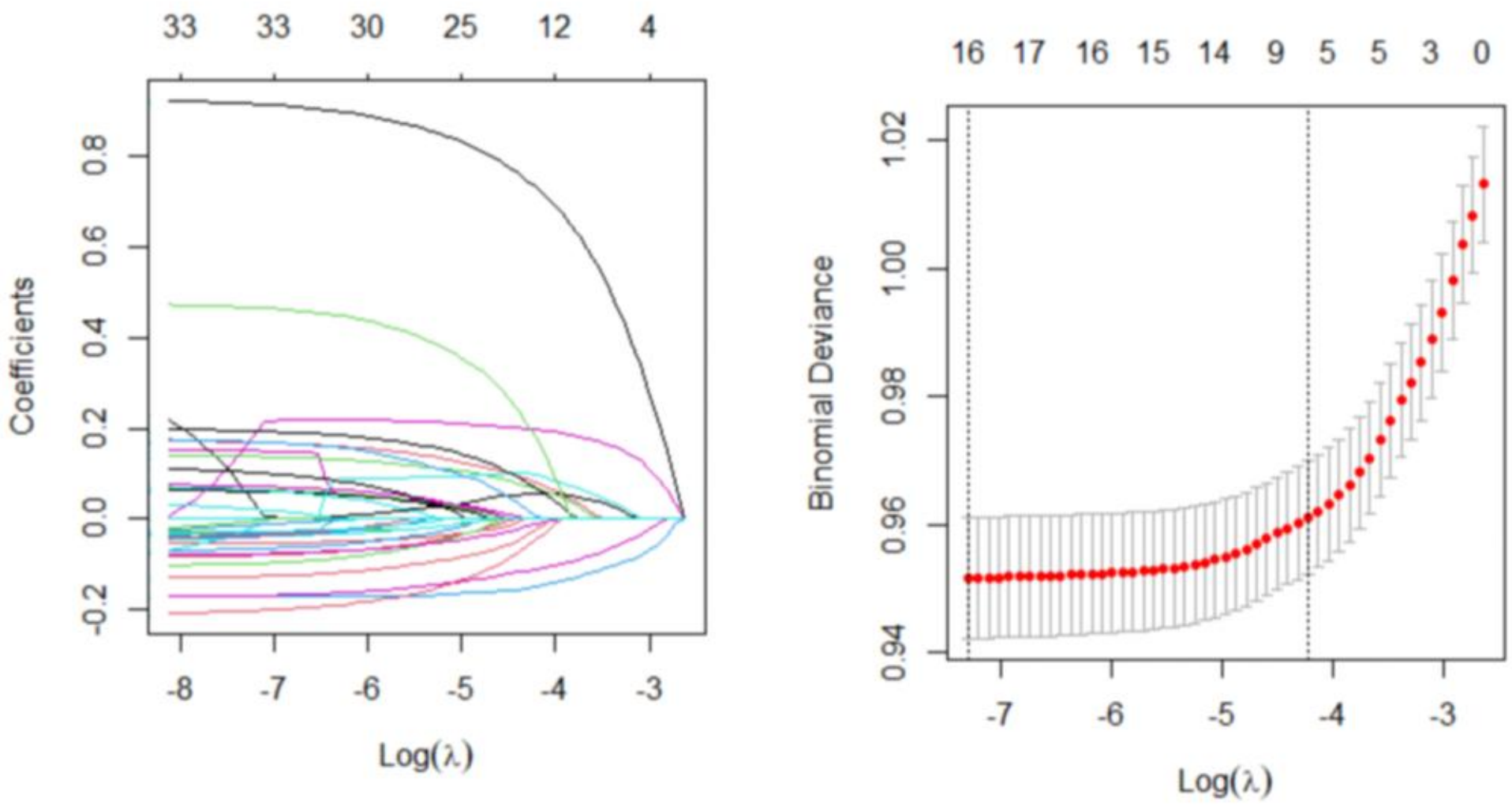

Figure S3.LASSO coefficient path diagram screening features and regression cross-validation curves. Panel A. Chinese growth standard. Panel B. WHO growth standard. LASSO – Least Absolute Shrinkage and Selection Operator, WHO – World Health Organization.

Abbreviations: LASSO, Least Absolute Shrinkage and Selection Operator.

Figure S4. Online application tool for the prediction of childhood and adolescent obesity assessed by Chinese (Panel A) and WHO (Panel B) standards.

A

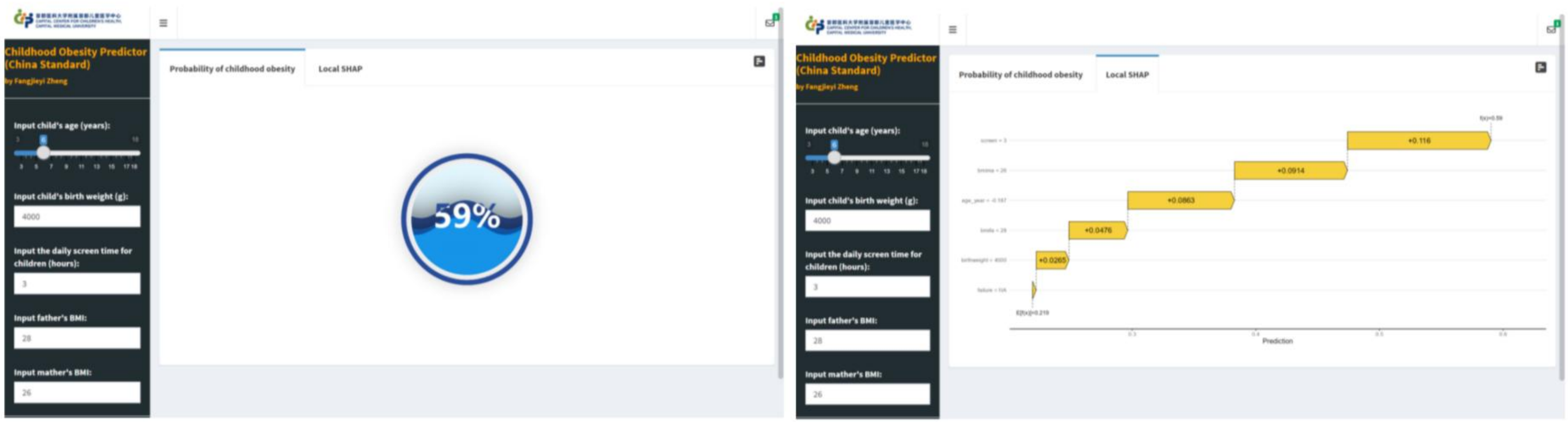

B

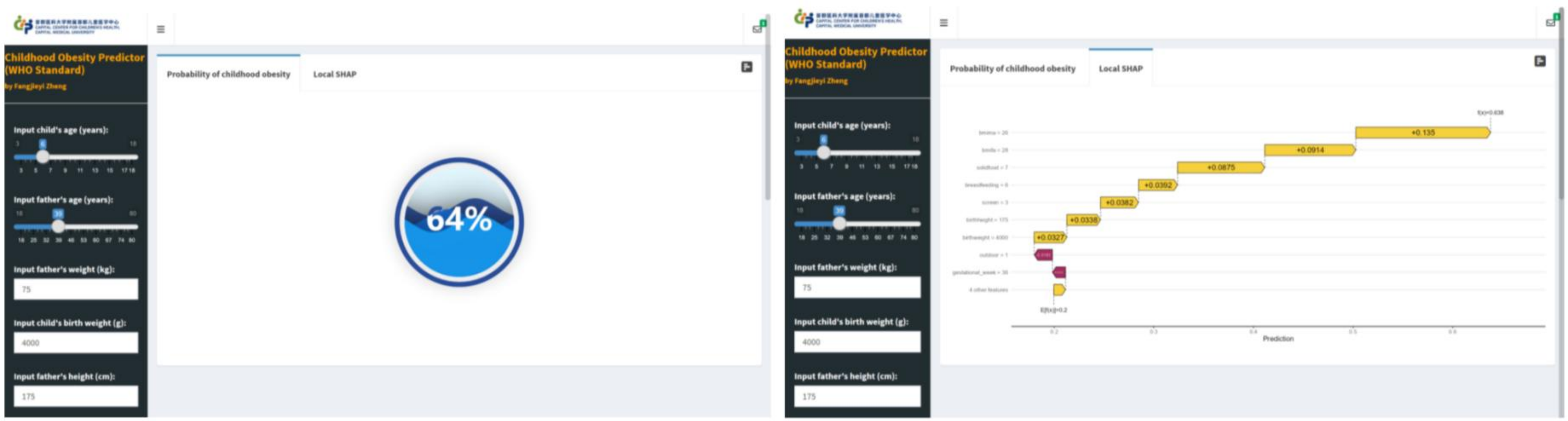

Supplement: Supplementary file 1 [file jogh-16-04299-s001.pdf]
